# Presumed Stroke in a Cat—A Case Report

**DOI:** 10.3390/vetsci12040320

**Published:** 2025-04-01

**Authors:** Sorin Marian Mârza, Radu Lăcătuș, Felix Daniel Lucaci, Lucia Bel, Ștefania Dandea, Iulia Melega, Robert Cristian Purdoiu

**Affiliations:** Faculty of Veterinary Medicine, Romania Department of Veterinary Clinical Sciences, University of Agricultural Sciences and Veterinary Medicine Cluj-Napoca, 400372 Cluj-Napoca, Romania; sorin.marza@usamvcluj.ro (S.M.M.); radu.lacatus@usamvcluj.ro (R.L.); lucia.bel@usamvcluj.ro (L.B.); stefania.dandea@usamvcluj.ro (Ș.D.); iulia.melega@usamvcluj.ro (I.M.); robert.purdoiu@usamvcluj.ro (R.C.P.)

**Keywords:** cat, brain, stroke, CT

## Abstract

This paper presents a detailed imaging-based approach to the diagnosis and evaluation of suspected ischemic stroke in a cat. The study describes a 12-year-old cat presented to our clinic with symptoms such as wheezing and inspiratory dyspnea, but without neurological or behavioral abnormalities. A computed tomography (CT) scan of the head revealed rhinitis, cerebral atrophy, and hypodense lesions in the right frontal and parietal brain regions, primarily supplied by the middle and rostral cerebral arteries. The lesion distribution, combined with cerebral atrophy and nonspecific clinical presentation, supported a provisional diagnosis of ischemic stroke. This case underscores the value of advanced imaging techniques in detecting incidental cerebrovascular events in feline patients, where initial respiratory symptoms, rather than overt neurological deficits, prompted investigation.

## Simple Summary

Ischemic stroke was identified in a feline patient that did not present any clinical or neurological signs of this condition. Computed tomography revealed cerebral atrophy and hypodense brain lesions in the right frontal and parietal regions. These lesions were located within the vascular territories of the middle and rostral cerebral arteries, consistent with ischemic stroke, despite the absence of magnetic resonance imaging (MRI) confirmation due to resource constraints. This case is notable for the lack of evident neurological signs at presentation, with subtle deficits detected only upon targeted examination.

## 1. Introduction

The term ‘stroke’ (or cerebrovascular accident) is a widely used, nonscientific descriptor for the sudden onset of localized, nonconvulsive, and nonprogressive brain symptoms resulting from vascular damage [1,2]. As the most frequent clinical manifestation of cerebrovascular disease—a condition compromising cerebral blood supply—stroke is conventionally defined as an intracranial vascular lesion with clinical signs persisting for at least 24 h. If symptoms resolve sooner, the event is classified as a transient ischemic attack [3]. This distinction is particularly relevant in feline patients, where imaging, as demonstrated in our case, is critical for confirming such diagnoses.

Cerebral vascularization in cats differs markedly from that in dogs, influencing lesion distribution patterns. In cats, the arterial circle of Willis is primarily perfused via anastomoses involving the maxillary and pharyngeal arteries, both originating from the external carotid artery. Unlike in dogs, the internal carotid artery in cats becomes non-functional shortly after birth. A distinctive feature of feline cerebral blood supply is the rete mirabile of the maxillary artery, a vascular network located in the pterygoid fossa. Formed by branches of the maxillary artery, it traverses the orbital fissure to enter the cranial cavity, contributing to the cerebral arterial circle alongside other cerebral arteries. However, the caudal brainstem is supplied by the vertebral arteries [4,5,6].

Pathologically, cerebrovascular lesions are classified into two main categories: ischemic stroke, caused by occlusion of a cerebral vessel by a thrombus or an embolus, resulting in reduced oxygen and glucose delivery to the brain, and hemorrhagic stroke, resulting from the rupture of a blood vessel within the brain parenchyma [1,7].

Feline ischemic encephalopathy may result from vasospasm in the middle cerebral artery, often following cerebrospinal migration of *Cuterebra* fly larvae. The resultant lesions are typically unilateral, affecting both white and gray matter within the cerebral hemispheres, predominantly in the vascular territory of the middle cerebral artery. Grossly, necrosis may be multifocal or extensive, affecting most of one hemisphere, with possible hemorrhages in the neuroparenchyma or leptomeninges. Histologically, findings include superficial laminar cortical necrosis with ischemic neuronal changes and, in some cases, frank parenchymal infarction. The disease may affect cats of any age, presenting clinical signs such as depression, ataxia, seizures, behavioral changes, and blindness, indicative of unilateral cerebral dysfunction. The diagnosis is based on the sudden onset of forebrain signs in cats, but a definitive diagnosis remains challenging even with post-mortem examination [8,9].

## 2. Case Description

A 12-year-old female European Shorthair cat, weighing 1.2 kg, was presented to our clinic for advanced diagnostic imaging. The primary reason for referral was the presence of respiratory symptoms, including wheezing and inspiratory dyspnea, which prompted a computed tomography (CT) scan of the head to investigate potential underlying causes. General clinical examination revealed a normothermic patient with stable respiratory and cardiac rates. The cat also exhibited bilateral mucous nasal discharge. Routine hematological analyses and serum biochemical testing yielded results within normal limits. A nasal swab test indicated that the patient was positive for *Pseudomonas* spp. with resistance to most antibiotics, except for amikacin.

To evaluate potential upper respiratory tract lesions, a CT scan of the head was performed under general anesthesia to ensure optimal image acquisition and minimize movement. Intramuscular premedication with a combination of alfaxalone 2 mg/kg (Alfaxan Multidose 10 mg/mL, Zoetis, NJ, USA), Butorphanol 0.2 mg/kg (Nalgosed 10 mg/mL, Bioveta, Ivanovice na Hane, Czech Republic), and ketamine 1 mg/kg (Narkamon Bio 10%, Bioveta, Czech Republic) facilitated intravenous access and induction with Propofol 4 mg/kg (Propofol-Lipuro 1%, B. Braun Melsungen, Melsungen, Germany), followed by endotracheal intubation with a cuffed ET tube. Throughout the procedure, anesthesia was maintained with isoflurane in oxygen (Isothesia 1000 mg/g, Covetrus, Voorschoten, The Netherlands) using a circle system.

Helical CT scanning of the head was obtained using a Siemens CT Somatom Scope machine with 16 channels, with the patient positioned in sternal recumbency. Head images were obtained in the axial plane using a 512 × 512 matrix, narrow windows (WW: 120, WL: 40), 3 mm slice thickness, and a pitch of 1.5. A multiplanar image reconstruction of the head was obtained using soft tissue and bone window algorithm at a slice thickness of 0.75 mm, and post-contrast images were obtained after the administration of 600 mg iodine/kg of iohexol (Omnipaque 350 mg/mL, GE Healthcare, Chicago, IL, USA) through a cephalic venous angio-catheter, using a power injector (Mallinckrodt LF Dual Head CT Injector).

**Figure 1:**
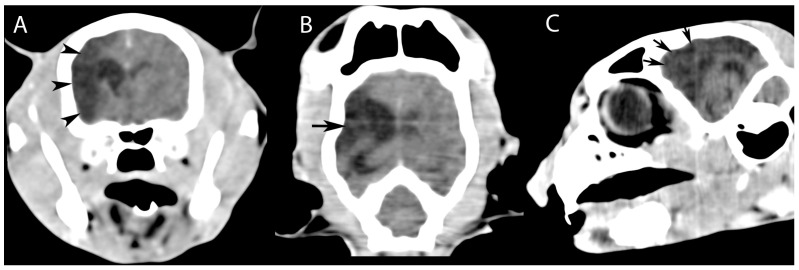
Post-contrast computed tomography (CT) images of the head, obtained in transverse, coronal, and sagittal planes, demonstrate the following findings: (**A**) transverse view: hypodense lesions localized within the brain parenchyma in the region anatomically corresponding to the right parietal bone (black arrowheads); (**B**) dorsal view: hypodense lesions of the brain parenchyma involving the right frontoparietal bone region (black arrow); and (**C**) sagittal view: hypodense lesions of the brain parenchyma identified in the region corresponding to the frontal bone (black arrows).

**Figure 2:**
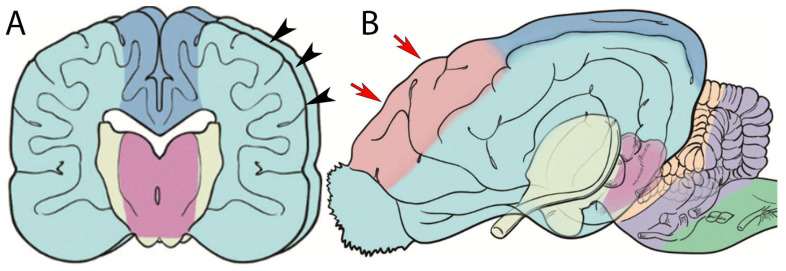
(**A**,**B**). Schematic draw of the vascular supply to the brain. Black arrowheads indicate the brain area that is supplied by the middle cerebral artery and red arrows indicate the area supplied by the rostral cerebral artery. Source: Scrivani, 2022 [10], modified.

CT images of the head revealed, in addition to rhinitis, a widespread region of hypoattenuation (17 HU mean) predominantly in the right hemisphere (Figure 1), corresponding to the vascular territory of the middle cerebral artery (Figure 2). This abnormality suggested reduced blood flow in the affected brain region, leading to tissue damage and the characteristic low-attenuation appearance on the scan. The right lateral ventricle was larger than the left one.

Since there were no neurological clinical signs observed by the owners and during the initial consultation only upper airway respiratory signs were observed, a neurological examination, prompted by CT findings, revealed subtle deficits such as mild left-sided postural response deficits, consistent with involvement of the right parietal lobe affecting motor and sensory pathways. The menace response was reduced in the left eye, while pupillary light reflexes remained intact. The cat exhibited mildly obstructive behavior, suggestive of forebrain dysfunction, but no seizure activity was observed or reported by the owners. These findings localized the lesion to the right anterior region; however, neurological signs remained subtle and were evident only upon a neurological examination.

## 3. Discussion

Current literature and research on cerebrovascular disease in feline patients remain relatively scarce. As mentioned earlier, the vascular architecture and hemodynamic patterns in feline cerebrovascular systems exhibit distinctive anatomical and physiological characteristics, which influence both the presentation and progression of cerebrovascular disorders in this species [4].

At the time of hospitalization, our patient showed no observable neurological or behavioral signs, a finding consistent with the existing medical literature. In a study by Altay et al., 2011 [4], which investigated sixteen feline patients diagnosed with cerebrovascular diseases, only six exhibited distinct neurological symptoms, while the remaining ten presented non-specific, extra-neurological manifestations, such as a decreased appetite or other generalized clinical signs.

Although the exact prevalence of cerebrovascular disorders in animals remains uncertain, data suggest that these conditions occur at a significantly lower rate in animals compared to humans [11]. In human medicine, the incidence of stroke has shown a notable increase over a decade, rising from 2.8% to 7.8% of the population, with most cases occurring in individuals over 65 years of age. In contrast, cerebrovascular diseases of non-traumatic origin appear considerably less common in veterinary patients, with only 3% of cats being diagnosed with such conditions over a six-year observation period, and between 1.5 and 2% of dogs studied in a referral hospital [4,12,13]. Previous studies suggest that the relatively lower incidence of cerebrovascular diseases in dogs, compared to humans, may be partly attributed to the differences in the cerebral blood supply. Specifically, dogs possess a greater number of intra- and extracranial arterial anastomoses, providing additional collateral circulation that may offer protection against ischemic events. This arterial network variation is not unique to dogs, and is also observed in cats [14]. One study reports that dogs surviving the initial phase of ischemic stroke generally have a fair to good prognosis, while 23% die within the first 30 days; those surviving beyond this period had a median survival time of 505 days [7]. Based on the authors’ knowledge, there is no study on the survival time in cats with stroke.

In feline patients, several concurrent diseases and predisposing factors have been identified as potential contributors to cerebrovascular disease, including parasitic migration caused by *Cuterebra* larvae, heartworm migration, ischemic events associated with anesthesia, intracranial telangiectasia, granulomatous meningoencephalitis, thiamine deficiency, and hypercoagulable states [4]. Some of these conditions are also recognized as significant risk factors for cerebrovascular disorders in human and canine patients [4]. For example, chronic hypertension is a well-established risk factor for cerebrovascular disease in humans, primarily due to its role in promoting atherosclerosis within small penetrating cerebral arteries. In our case, no cardiological evaluation or blood pressure measurement was performed, due to the absence of systemic clinical signs suggestive of hypertension or cardiac disease at presentation. However, systemic hypertension, observed in cases of feline renal dysfunction, could contribute to cerebrovascular risk, and warrants consideration in future assessments [15].

CT findings in ischemic stroke vary depending on the size and location of the affected vessel and the time elapsed since infarction onset. In acute ischemic stroke, typically observed 3–6 h post-symptom onset, a subtle reduction in parenchymal density is accompanied by a mild mass effect due to cerebral edema. Post-contrast imaging, performed 24 h to one week after infarction, may reveal peripheral enhancement. In the chronic phase, ischemic strokes exhibits progressively well-defined margins around the infarcted region, ultimately resulting in a noticeable reduction in parenchymal volume [16]. Typically, a presumptive diagnosis relies on clinical suspicion supported by MRI findings. Hyperintensities on T2-weighted (T2W) images, indicative of edema, may be observed in the frontal and parietal lobes, corresponding to the vascular territory supplied by the middle cerebral artery [17]. Although MRI offers a greater sensitivity than CT for detecting ischemic stroke and subtle cerebral changes, it was not performed in this case due to limited availability and the patient’s stable condition, which did not necessitate immediate escalation beyond CT [18].

Differential diagnoses for the observed lesions in our patient included several potential causes based on their characteristic imaging features. Vasogenic edema, as visualized on CT scans, typically appears as regions of mild to moderate hypoattenuation compared to the surrounding normal brain parenchyma, without a specific localization, and may cause a mass effect. This edema results from the disruption of the blood–brain barrier, leading to extracellular fluid accumulation. Another differential was ex vacuo hydrocephalus, which commonly occurs secondary to cerebral atrophy, often following a traumatic brain injury or a neurodegenerative process and is characterized by a compensatory passive dilation of the lateral ventricles due to brain tissue loss rather than increased intracranial pressure [19]. Feline ischemic encephalopathy may affect the frontal lobes, but is typically observed only in younger patients [10].

**Figure 3:**
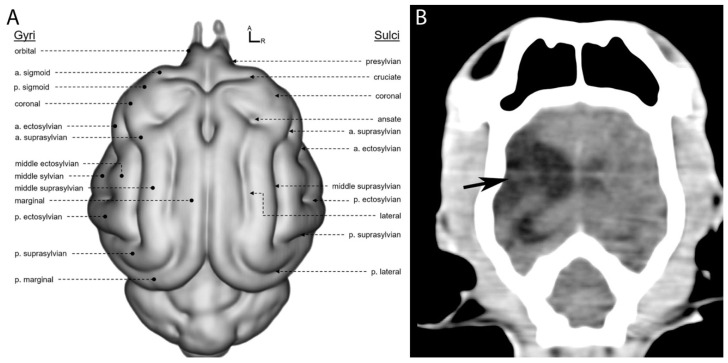
Coronal view of the feline brain. (**A**) Anatomical features of the gray matter of the cat brain and (**B**) a dorsal view of the CT scan of the patient (original). Source: (**A**) Stolzberg et al., 2017 [20], modified.

The approximate location of brain lobes in carnivores can be determined based on the position of the corresponding frontal, parietal, and occipital bones of the calvaria. These cerebral lobes are typically situated directly beneath the bones of the same name. However, distinguishing these bones is often challenging in imaging, particularly in transverse CT scans [10]. The hypodense areas observed in the right hemisphere on the CT scan correspond anatomically to regions primarily involving the frontal and parietal lobes (Figure 3), as shown in the anatomical diagram (Figure 3A). Specifically, the lesions were presumed to be located in the anterior suprasylvian gyrus and middle ectosylvian gyrus (part of the frontal lobe) and the posterior suprasylvian sulcus (part of the parietal lobe).

**Figure 4:**
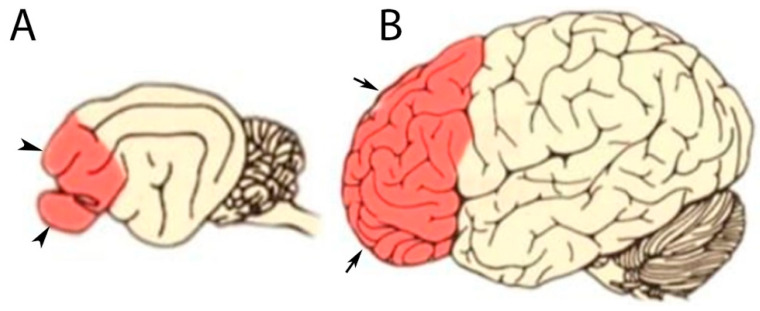
Comparison of prefrontal cortex sizes between (**A**) cats (arrowheads) and (**B**) humans (arrows). Source: Valencia-Ortiz et al., 2020 [24] modified.

In cats, the frontal lobes, constituting only 3 to 3.5% of the total brain, are smaller than in humans, where they account for nearly 25% of the brain (Figure 4). In cats, the frontal lobes are responsible, among other functions, for analyzing odors, regulating temperament, and facilitating social skills and certain movements, while the parietal lobe, particularly the middle suprasylvian cortex, is essential for processing visual motion and managing attentional functions. This region plays a key role in detecting and analyzing motion direction and enabling orientation toward new visual stimuli in the field of view [21,22,23]. We hypothesize that the relatively smaller and less complex frontal lobe in cats likely contributes to a less pronounced expression of cerebrovascular pathology compared to humans, where such lesions often manifest as marked neurological deficits [12].

Computed tomography plays an important role in the diagnostic assessment of cerebrovascular disease by providing valuable imaging that reveals a wide range of pathophysiological changes associated with stroke. In the acute phase, CT imaging can identify early alterations in tissue attenuation and subtle mass effects. However, while CT is effective in these contexts, it may be less capable of fully detecting ischemic and lacunar infarctions compared to MRI, which offers greater sensitivity for these stroke types [18].

## 4. Conclusions

Considering all the details, the observed lesion exhibits a distinct distribution pattern, characterized by a well-defined hypodense area within the vascular territory of the middle and rostral cerebral arteries. This finding is accompanied by evidence of cerebral atrophy, suggestive of chronic ischemic changes. The hypodensity and associated structural alterations indicate a disrupted cerebral perfusion, likely resulting from an ischemic event. These imaging characteristics strongly align with the pathophysiological features of a cerebral stroke, supporting a presumptive diagnosis of an ischemic cerebrovascular accident.

Despite the evident brain lesions, the cat showed no clinical or behavioral changes detectable during the clinical examination or reported by the owner.

A neurological examination, recommended following the imaging assessment, revealed minimal and very subtle neuronal deficits.

## Data Availability

This article includes all relevant study data.

## Abbreviations

The following abbreviations are used in this manuscript:

CT	computed tomography
CVD	cerebrovascular disease
HU	Hounsfield units
KG	kilogram
MG	milligrams
MRI	Magnetic Resonance
WL	window level
WW	window width

## References

[1] Garosi L.S. (2010). Cerebrovascular disease in dogs and cats. Vet. Clin. N. Am. Small Anim. Pract..

[2] Cherubini G.B., Rusbridge C., Singh B.P., Schoeniger S., Mahoney P. (2007). Rostral cerebellar arterial infarct in two cats. J. Feline Med. Surg..

[3] Wessmann A., Chandler K., Garosi L. (2009). Ischaemic and haemorrhagic stroke in the dog. Vet. J..

[4] Altay U.M., Skerritt G.C., Hilbe M., Ehrensperger F., Steffen F. (2011). Feline cerebrovascular disease: Clinical and histopathologic findings in 16 cats. J. Am. Anim. Hosp. Assoc..

[5] Negrin A., Taeymans O.N.J., Spencer S.E., Cherubini G.B. (2018). Presumed Caudal Cerebellar Artery Infarction in Three Cats: Neurological Signs, MRI Findings, and Outcome. Front. Vet. Sci..

[6] Korim F., Kuricová M., Vdoviaková K., Krešáková L. (2023). Fascinating wonderful network: Rete mirabile of the maxillary artery in cats—Minireview. Vet. Res. Commun..

[7] Gredal H., Toft N., Westrup U., Motta L., Gideon P., Arlien-Søborg P., Skerritt G.C., Berendt M. (2013). Survival and clinical outcome of dogs with ischaemic stroke. Vet. J..

[8] Zachary J.F. (2022). Pathologic Basis of Veterinary Disease.

[9] Hazenfratz M., Taylor S.M. (2018). Recurrent seizures in cats: Diagnostic approach—When is it idiopathic epilepsy?. J. Feline Med. Surg..

[10] Scrivani P.V. (2022). Veterinary Head and Neck Imaging.

[11] Tidwell A.S., Mahony O.M., Moore R.P., Fitzmaurice S.N. (1994). Computed tomography of an acute hemorrhagic cerebral infarct in a dog. Vet. Radiol. Ultrasound.

[12] Imoisili O.E., Chung A., Tong X., Hayes D.K., Loustalot F. (2024). Prevalence of Stroke—Behavioral Risk Factor Surveillance System, United States, 2011–2022. Morb. Mortal. Wkly. Rep..

[13] Arnold S.A., Platt S.R., Gendron K.P., West F.D. (2020). Imaging Ischemic and Hemorrhagic Disease of the Brain in Dogs. Front. Vet. Sci..

[14] Whittaker D.E., Drees R., Beltran E. (2018). MRI and clinical characteristics of suspected cerebrovascular accident in nine cats. J. Feline Med. Surg..

[15] Boudreau C.E. (2018). An Update on Cerebrovascular Disease in Dogs and Cats. Vet. Clin. N. Am. Small Anim. Pract..

[16] Schwarz T., Saunders J. (2011). Veterinary Computed Tomography.

[17] Lorenz M.D., Coates J.R., Kent M. (2011). Handbook of Veterinary Neurology.

[18] Paul A.E., Lenard Z., Mansfield C.S. (2010). Computed tomography diagnosis of eight dogs with brain infarction. Aust. Vet. J..

[19] Wisner E.R., Zwingenberger A.L. (2015). Atlas of Small Animal CT and MRI.

[20] Stolzberg D., Wong C., Butler B.E., Lomber S.G. (2017). Catlas: An magnetic resonance imaging-based three-dimensional cortical atlas and tissue probability maps for the domestic cat (*Felis catus*). J. Comp. Neurol..

[21] Forrest D.V. (2002). The Executive Brain: Frontal Lobes and the Civilized Mind. Am. J. Psychiatry.

[22] Kim J. Cat Brain Anatomy: Vet-Verified Facts & Common Disorders. https://www.catster.com/cat-health-care/cat-brain-anatomy/.

[23] Lomber S.G., Payne B.R., Cornwell P., Long K.D. (1996). Perceptual and cognitive visual functions of parietal and temporal cortices in the cat. Cereb. Cortex.

[24] Valencia-Ortiz A.I., Consuelos-Barrios M., Garcia-Cruz R., García-López E. (2020). Orbitofrontal cortex and aggressive behavior in children ages 11 to 13. J. Basic Appl. Psychol. Res..

